# Concomitant Central Nervous System Toxoplasmosis and Seronegative Disseminated Coccidioidomycosis in a Newly Diagnosed Acquired Immune Deficiency Syndrome Patient

**DOI:** 10.1177/2324709619869372

**Published:** 2019-08-17

**Authors:** Michael Valdez, Leila Moosavi, Arash Heidari

**Affiliations:** 1 Kern Medical—UCLA, Bakersfield, CA, USA

**Keywords:** CNS toxoplasmosis, disseminated, miliary pneumonia, coccidioidomycosis, fungemia, seronegative

## Abstract

Opportunistic infections (OIs) are a significant cause of morbidity and mortality in immunosuppressed patients and may be due to bacteria, virus, protozoa, or fungi. Toxoplasmosis is a common cause of central nervous system infection in human immunodeficiency virus (HIV) patients. Coccidioidomycosis is a relatively common fungal infection that may lead to disseminated disease and fungemia in immune-compromised hosts living in endemic regions. This single-patient case report documents the presentation, diagnosis, management, and outcome of concomitant central nervous system toxoplasmosis and diffuse miliary pneumonia with fungemia due to disseminated seronegative *Coccidioides immitis* in a 33-year-old male patient recently diagnosed with chronic advanced HIV. Impaired cellular immune function, such as defects in the IL-12/IFN-γ pathway or T-helper IL-17-mediated response, is associated with increased severity of coccidioidomycosis. Fungemia and acute respiratory distress syndrome are both associated with very high mortality in coccidioidomycosis. In HIV hosts, negative *Coccidioides* serology can be seen in up to 25% of cases and therefore other diagnostic modalities should be initiated promptly and simultaneously. This case demonstrates simultaneous OI in the setting of advanced acquired immune deficiency syndrome and emphasizes the need for early diagnosis of HIV and OI in order to ensure prompt initiation of antiretroviral therapy, prophylactic, and therapeutic medications.

## Introduction

Opportunistic infections (OIs) are infections that occur with greater frequency and increased severity in immune-compromised hosts.^1,2^ Bacteria, virus, fungi, or protozoa are responsible for causing OIs.^1^ Prevalence of OI is inversely proportional to CD4 count.^1,2^ Toxoplasmosis is a common central nervous system (CNS) infection in HIV patients with seroprevalence of 11% in the United States.^1^ One study conducted during the pre–antiretroviral therapy (ART) era states that toxoplasmosis was the most common CNS OI in AIDS patients.^3^ Coccidioidomycosis is a relatively common fungal infection that may lead to diffuse reticulonodular pneumonia, fungemia, and disseminated diseases in immune-compromised hosts residing in endemic locations.^4^ Immunosuppressed patients are at increased risk of disseminated coccidioidomycosis due to impaired cellular immune function.^5,6^ The regular use of ART has been critically important in preventing OI, with one study reporting a decrease in rate of OI in HIV patients from 140 per 1000 person/years in 1995 to <20 per 1000 person/years in 2007.^2,7,8^ In this article, we describe a fatal case of disseminated coccidioidomycosis and CNS toxoplasmosis in a 33-year-old Hispanic male with newly diagnosed AIDS.

## Case Report

A 33-year-old Hispanic male with unremarkable past medical history presented to another hospital with headaches for 1.5 weeks associated with blurring of vision. He denied any recent illnesses or other symptoms prior to the onset of headaches. Magnetic resonance imaging (MRI) of the brain revealed a 2.7-cm ring-enhancing intracranial lesion in the right temporal lobe with mass effect. He was subsequently transferred to our facility for neurosurgical intervention. After right craniotomy with mass resection and biopsy, the patient became febrile. On initial workup, he was screened and diagnosed with AIDS; CD4 count was less than 20 cells/µL (1%) and RNA-PCR (polymerase chain reaction) 191 000 copies/mL. ART, azithromycin 1200 mg PO (per os) once weekly for *Mycobacterium avium* complex prophylaxis, and trimethoprim/sulfamethoxazole 400 mg intravenous Q12H (every 12 hours) for treatment of presumed CNS toxoplasmosis were started. Further investigation revealed that the patient was made aware of HIV diagnosis 2 years prior but remained in denial.

Comprehensive screening in this immunocompromised host revealed elevated immunoglobulin (Ig) G for toxoplasma, positive serum *Cytomegalovirus* IgG, positive serum herpes simplex virus-1 IgG and herpes simplex virus-2 IgG, and reactive hepatitis A antibody. *Coccidioides* serology was nonreactive for IgM and IgG with complement fixation (CF) titer <1/2. Lumbar puncture showed cell count 48 cells/µL, RBC 7 cells/µL, neutrophils 0%, lymphocytes 97%, glucose 55 mg/dL, protein 42 mg/dL, and opening pressure 210 mm H_2_O. All cerebrospinal fluid (CSF) studies, including *Coccidioides* serology, aerobic and fungal cultures, cryptococcal antigen screen, and acid-fast *Bacillus* (AFB) smear/culture, were negative except for positive CSF toxoplasma IgG with DNA-PCR for *Toxoplasma gondii* 286 copies/mL. HIV-1 subtype B was identified with no predicted genotypic resistance to reverse transcriptase inhibitors, protease inhibitors, or integrase inhibitors. Screening for HLA-B5701 was negative. Single-tablet regimen of abacavir/dolutegravir/lamivudine was selected as the preferred regimen for ART.

The hospital course was complicated as the patient remained persistently febrile with temperatures up to 39.4°C. Blood cultures were negative without leukocytosis or bandemia. Empiric therapy for brain abscess with ceftriaxone, vancomycin, and metronidazole was initiated. MRI of the brain on postoperative day 4 showed a reduction in the size of the ring-enhancing lesion with no mass effect and appearance consistent with toxoplasmosis. Immunostains from the biopsy specimens showed bradyzoites and tachyzoites consistent with toxoplasmosis and confirmed the presumed diagnosis. Periodic acid–Schiff and Gomori methenamine silver stains were negative for fungal organisms and AFB stain was negative for acid-fast bacilli. No evidence of malignancy was identified. Antibiotics for possible brain abscess were discontinued. The patient was discharged after clinical symptoms improved and fever resolved. Discharge medications included trimethoprim/sulfamethoxazole, azithromycin, and abacavir/dolutegravir/lamivudine.

Approximately 2 weeks later, the patient represented to the emergency department with fevers, generalized weakness, and 1-day history of cough productive of white sputum. The temperature was 39.6°C and chest X-ray (CXR) showed a new area of left upper lobe (LUL) opacification with diffuse reticulonodular prominence of the interstitium. Computed tomography scan of brain without contrast showed postsurgical changes in the right temporal lobe with no mass effect or intracerebral hemorrhage. Broad-spectrum antibiotics with vancomycin and piperacillin/tazobactam plus fluconazole were started and the patient was placed on airborne precautions until tuberculosis could be ruled out. Repeat MRI showed no irregular enhancing lesions, and *Coccidioides* serology was again negative.

**Figure 1.**
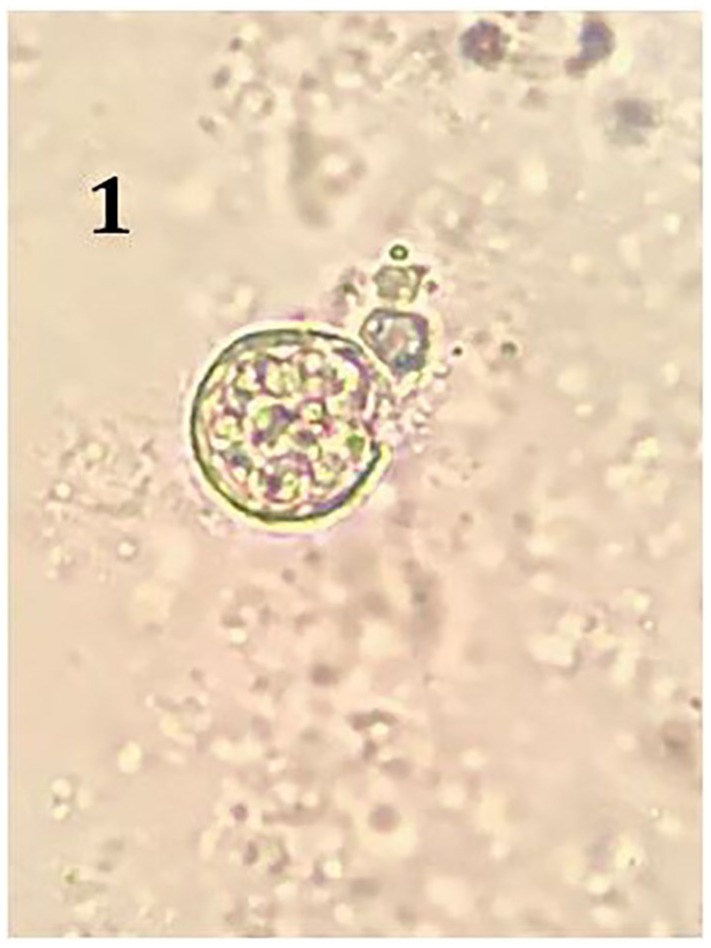
Spherule containing endospores on potassium hydroxide wet mount from bronchoalveolar lavage.

**Figure 2.**
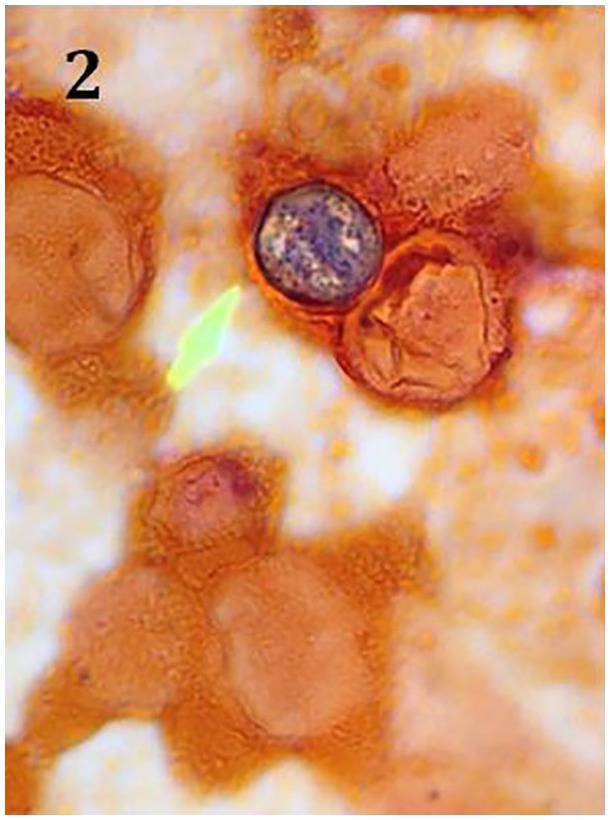
Spherule containing endospores on gram stain from bronchoalveolar lavage.

**Figure 3.**
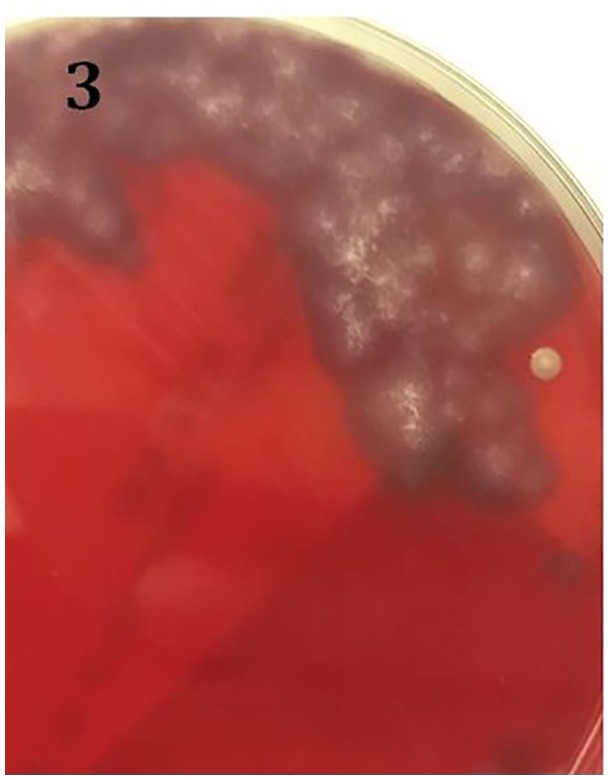
Plate from blood culture with *Coccidioides immitis* growth. As infectious spores may be easily disseminated by air currents, *Coccidioides* poses a significant laboratory hazard. This photograph was obtained through the glass of a safety cabinet in which the culture plate cover remained in place.

The patient’s condition continued to deteriorate. Repeat CXR showed diffuse infiltrates with air bronchograms, ground glass opacities, and consolidation much worse relative to the prior examination. Computed tomography scan of chest revealed extensive reticulonodular interstitial infiltrates with focal consolidation in the LUL. Bronchoscopy was arranged but the patient required intubation due to worsening hypoxemia. After 3 sputum AFB smears were negative, bronchoalveolar lavage was performed and histopathology from the LUL showed spherules containing endospores on potassium hydroxide wet mount (Figure 1) and gram stain (Figure 2). Multiple blood cultures grew *Coccidioides immitis* (Figure 3). Antifungal treatment was changed to liposomal amphotericin B; however, the patient developed severe acute respiratory distress syndrome (ARDS) with fraction of inspired oxygen requirement of 60%. Electrocardiogram revealed tachycardia up to 140 beats per minute with wide QRS complexes. The patient went into cardiac arrest and subsequently expired.

## Discussion

The differential diagnosis for a ring-enhancing lesion in an HIV patient is broad, including CNS toxoplasmosis, primary CNS lymphoma, brain abscess, tuberculoma, and fungal abscess due to *Cryptococcus, Histoplasma*, or *Coccidioides*. After extensive workup including lumbar puncture and brain biopsy, CNS toxoplasmosis was confirmed in this particular case.

Toxoplasmosis is one type of OI, caused by the protozoan parasite *Toxoplasma gondii*, and is a common CNS infection in HIV/AIDS patients.^3,9,10^ It has a global distribution with prevalence ranging from 11% in the United States to >80% in a number of countries.^1^ Immunosuppression and prior infection are the most important risk factors with one study demonstrating 28% probability of CNS toxoplasmosis in seropositive patients not on ART or prophylactic medications with CD4 count <100 cells/µL.^2,9^

A presumptive diagnosis of toxoplasmosis in HIV patients is made when CD4 count is <100 cells/µL and serum IgG is positive, ring-enhancing lesions are present on MRI brain, and the clinical presentation includes fever, headaches, and neurological deficits.^1,10^ Despite low sensitivity, positive CSF DNA-PCR for *Toxoplasma gondii* has reported >96% specificity for toxoplasmosis and definitive diagnosis can be made with biopsy showing tachyzoites.^11,12^ Treatment is generally started based on presumptive diagnosis as brain biopsy has been linked with increased morbidity and mortality.^10,11,13^ Sulfadiazine, pyrimethamine, and leucovorin is the preferred treatment regimen but trimethoprim/sulfamethoxazole, as was administered in this patient, is an acceptable alternative.^14^ The benefit of prophylaxis has been demonstrated as the risk of developing toxoplasmosis was 0% to 2.4% in one particular study where HIV patients received prophylactic therapy with trimethoprim/sulfamethoxazole.^15^

Coccidioidomycosis is an opportunistic fungal infection that is well reported in immune-compromised hosts and may be due to either reactivation of prior infection or newly acquired infection.^16,17^ In addition to living in an endemic area, HIV patients are more susceptible due to impaired cellular immune function, including defects in the IL-12/IFN-γ pathway and T-helper IL-17-mediated response.^6^ Clinical diagnosis of AIDS and CD4 count <250 cells/µL are the most critical factors contributing to the development active coccidioidomycosis.^5,16^

In this particular case, the rapid progression of CXR findings was unique. CXR during the final admission showed a new LUL lesion and reticulonodular interstitial infiltrates consistent with miliary coccidioidomycosis that were not present on studies 2 weeks prior. Serologic testing for IgM and IgG antibodies with CF is regularly used in order to diagnose infections due to *Coccidioides* and help guide therapy.^4,18^ One small study, analyzing patients with HIV and disseminated *C immitis*, showed consistently negative CF titers in 25% of patients.^18^ Another retrospective study documents initial negative serology in 17% of HIV patients with confirmed *Coccidioides*.^19^ The repeatedly negative CF titers in this case, despite miliary disease with cocci spherules in bronchoalveolar lavage and fungemia with cultures growing *C immitis*, demonstrate that in HIV patients the usefulness of CF titers remains unknown and methods such as histopathology or culture are essential in identifying a definitive diagnosis.^18-20^

Diffuse pulmonary processes are associated with unfavorable prognoses and are most commonly identified in advanced cases of immunosuppression, like the CD4 count <20 cells/µL in our patient.^4,19,21^ Dyspnea and fever are the most common symptoms and the reticulonodular pattern revealed by imaging is generally attributed to hematogenous spread in patients with fungemia.^22^ Additional modalities that aid in diagnosis include histopathology showing spherules and fungal cultures.^20^ Amphotericin B is the preferred initial therapy for miliary pneumonia due to *Coccidioides* with a potential need for lifelong azole treatment.^4,23^ ARDS is one of the major complications that may arise from miliary pneumonia, with one study reporting nearly 100% mortality in immunosuppressed patients.^4^ Another study demonstrated that patients with fungemia experienced higher mortality rates within 1 month of positive culture relative to patients identified with *C immitis* infections without fungemia.^22^

## Conclusion

Opportunistic infections are one of the major factors contributing to morbidity and mortality in immunosuppressed hosts. Toxoplasmosis is a common cause of CNS infection in HIV patients, and coccidioidomycosis is a common fungal infection that may lead to disseminated disease in immune-compromised patients living in endemic areas. Fungemia and ARDS are both associated with very high mortality in coccidioidomycosis. Impaired cellular immune function is associated with increased severity of coccidioidomycosis, and in HIV hosts, negative serology can be seen in up to 25% of cases. Despite prior HIV diagnosis, our patient did not regularly follow-up with a primary physician or specialist. Subsequently, CD4 count was <20 cells/µL with AIDS-defining illness already present at the time of presentation to our facility. This case emphasizes the importance of eliminating barriers to care, such as unidentified financial burdens, unaddressed mental health needs, or stigma associated with AIDS in order for early diagnosis and treatment of both HIV and OI. This case demonstrates that in the setting of advanced AIDS, multiple OIs can occur simultaneously. In circumstances where a patient fails to improve clinically, initiation of additional diagnostic modalities is imperative in attempt for early diagnosis of OIs as well as prompt initiation of the appropriate treatment regimens in order to improve long-term prognosis and enhance quality of life.
